# Integrated Care in Switzerland: Strengths and Weaknesses of a Federal System

**DOI:** 10.5334/ijic.5668

**Published:** 2021-10-29

**Authors:** Séverine Schusselé Filliettaz, Peter Berchtold, Ursula Koch, Isabelle Peytremann-Bridevaux

**Affiliations:** 1 La Source School of Nursing, HES-SO University of Applied Sciences and Arts Western Switzerland, Av. Vinet 30, 1004 Lausanne, Switzerland; 2 Association for the Promotion of Integrated Patient Care Networks (PRISM), Geneva, Switzerland; 3 Forum Managed Care (FMC), Zugerstrasse 193, 6314 Neuägeri, Switzerland; 4 College M, Laupenstrasse 7, 3001 Bern, Switzerland; 5 Centre for Primary Care and Public Health (Unisanté), University of Lausanne, Route de la Corniche 10, 1010 Lausanne, Switzerland

**Keywords:** MeSH, Switzerland, integrated delivery systems, healthcare systems, political systems, health policies

## Abstract

**Introduction:**

Switzerland’s fragmented healthcare system mirrors its federal structure and mix of cultures and languages. Although the Swiss have a higher life expectancy than most of their neighbours, their healthcare system faces similar challenges that call for more integrated care (IC).

**Aim/Method:**

This article aims to provide insight into the specificities of and latest developments in Switzerland’s healthcare system and how they may have influenced the development and implementation of IC there.

**Description/Discussion:**

The number of local IC initiatives has been growing steadily for 20 years. With a certain lag, various policies supporting IC have been established. Among them, a recent democratic debate on the federal mandatory health insurance law could either induce a radical move towards centralised support for IC or continue to support scattered local IC initiatives.

**Conclusion:**

In the future, Switzerland’s healthcare system will probably navigate between local IC initiatives and centralised, federal support for IC initiatives. This will be the reflection of a very Swiss way forward in a world without clear evidence on whether centralised or decentralised initiatives are more successful at developing IC.

## Introduction

Switzerland’s slightly more than 8.5 million inhabitants live in the heart of Europe, unevenly separated into three main linguistic regions [1]. Swiss residents have a high life expectancy compared to other Europeans (e.g. 85.6 years for women in 2020) [1], and they are globally satisfied with the accessibility and quality of their care [2,3]. However, as in other European countries [4], the healthcare system is under pressure due to rising costs (11.2% of GDP in 2018) [2], the growing prevalence of non-communicable diseases and multimorbidity [5,6], patients’ increasing needs and expectations [3], and expected shortages of qualified professionals [7]. Enhancing integrated care (IC) in Switzerland could alleviate this pressure.

Switzerland is a federal state in which government health responsibilities are split between the national, cantonal and municipal levels [1]. The federal level regulates areas such as financing (e.g. mandatory health insurance), the quality and safety of pharmaceuticals and medical devices, public health (e.g. control of infectious diseases), and education and training [2]. Each of the 26 cantons, with its own minister of health, is responsible for licensing its healthcare providers, regulating its hospital services and subsidizing its healthcare institutions (e.g. in-patient, homecare) [2]. Each canton can be considered to have a slightly different healthcare system [8]. The 2202 municipalities are mainly responsible for long-term care and social services [2]. Finally, thanks to its system of direct democracy, Swiss citizens are involved in shaping new laws or adjusting existing ones at all three levels, in addition to electing representatives [8]. These elements highlight the Swiss health system’s configuration of centralized and decentralized responsibilities for the building blocks of IC [9].

In Switzerland, universal access to healthcare was established through a mandatory health insurance (MHI) introduced in 1994 [10]. Following a principle of managed competition allowing citizens a free choice of health insurers and providers, the MHI aims to i) strengthen solidarity between healthy and sick people, ii) ensure high-quality healthcare and iii) contain costs [11,12]. The basic MHI coverage includes a comprehensive package of benefits with direct and unrestricted access to healthcare services. However, around 70% of the Swiss population opt for a cheaper, alternative insurance model including gatekeeping [13]. The not-for-profit MHI scheme is operationalised by more than 50 insurers, which also operate for-profit complementary health schemes [2].

In addition to MHI, healthcare services have other funding streams, such as out-of-pocket payments, direct and indirect public funding, and other social insurance schemes. These streams vary [14] between the numerous providers active in Switzerland. For instance, medical and non-medical providers of outpatient care are mainly reimbursed on a fee-for-service basis, and providers working in networks or health maintenance organisations (HMOs) are increasingly paid by salary and/or capitation [2]. These outpatient costs to the citizen are essentially covered by the MHI and out-of-pocket payments. On the contrary, inpatient care relies on a national diagnosis-related group (DRG) payment system [15], covered by both MHI and public funding. All health professionals are free to provide care and treatments that are covered by MHI. Except for some benchmarking used to monitor providers’ incomes (MHI law, art. 32 and 57), consistent and continuous quality controls have only recently emerged (e.g. patient outcomes [16]). Similarly, healthcare data transparency is still in its infancy [17].

Healthcare in Switzerland is provided by an array of private and public providers, ranging from individual to group practices and specialised institutions (e.g. homecare, long-term care), to large hospitals and health networks [2]. Additionally, providers are scattered geographically, housed in distinct buildings, and administratively separate and different (e.g. primary care medical networks and homecare institutions have different hierarchies). Moreover, although the number of multi-professional outpatient structures is increasing [18], many remain mono-professional and answer specific patients’ needs (e.g. homecare with nurses, physiotherapy practices). Regarding outpatient physicians, 48% worked in individual practices [19], and 55% were members of a physicians network in 2020 [20]. Furthermore, 75% of the general practitioners and 41% of the specialists were affiliated to such networks in 2020 [20]. Finally, new fields of work are emerging in these networks, such as triage and coordination by Advanced Practice Nurses [21].

This article aims to provide some insight into the specificities of and latest developments in Switzerland’s healthcare system and to discuss their influence on the development and implementation of IC.

## Latest key policy developments

Over the past ten years, several programmes and policies have directly or indirectly favoured the development of IC in Switzerland. They are presented chronologically and succinctly described below.

### 2013–2019: National dementia [22] and palliative care [23] strategies

Endorsed by both the national and cantonal governments, these strategies aimed to develop common frameworks for managing these conditions. The strategies highlighted the specific professional expertise available but also the limited incentives for collaboration and coordination. Their recommendations included specific focuses on the continuity of care and on an optimised framework for interprofessional coordination and networking providers. Based on this, several cantons developed their own policy, and the recommendations above were included in subsequent national policies (see below).

### 2015: New models for primary care [24]

In 1998, a National Health Policy Dialogue was initiated by the national and cantonal governments to iteratively address the challenges facing Switzerland’s healthcare system. The resulting 2015 report on New Models for Primary Care called for IC and a more global approach to patients’ needs. Building upon the Chronic Care Model [25], this report also highlighted the needs to improve interprofessional collaboration, optimise tasks and competencies, and adjust contextual elements, such as working conditions and education.

### 2015–2020: National research programme [26]

The Smarter Healthcare research programme, launched by the Swiss National Science Foundation, included 34 projects aimed at providing fresh insights and potential improvements to healthcare structures and utilisation. The synthesis of this programme is expected in 2022, and policy briefs will be issued in six areas: quality; patient choice; coordination and care models; costs and reimbursement; healthcare data; and communities of researchers, decision-makers and practitioners.

### 2015 onwards: National project on the coordination of care [27]

This project aims to enhance the quality of care by focussing on improved processes for meeting patient’s various needs and ensuring the continuity of care. Without explicitly saying so, it is an attempt to shift from *disease management*, focussing on health outcomes, towards *care/case management*, focussing on care processes—in line with recommendations from the literature on multimorbidity [28]. This project led to several reports addressing, e.g. improved transitions between inpatient and outpatient care [29] and the remuneration of coordination activities [30].

### 2017–2020: Promotion of interprofessional collaboration in healthcare [31]

Under the umbrella of a larger initiative tackling shortages of qualified healthcare professionals, this programme intended to foster the coordination of care and interprofessional collaboration. Good practice models were identified in educational as well as clinical settings [32,33]. Additionally, recommendations highlighted the needs for quality indicators, support for implementation, legal clarifications about the responsibilities of non-medical actors, improved funding and increased training [34].

### 2017–2024: National strategy to tackle non-communicable diseases [35]

This strategy aims to redirect the healthcare system towards the prevention and management of chronic conditions. To this end, it strengthens health promotion and prevention, and it is also designed to improve health literacy, especially of vulnerable populations. Furthermore, it considers various health determinants and promotes inter-sectoral collaboration (e.g. business, urban planning). Innovatively, it prompts federal and cantonal synergies in prevention.

### 2018–2022: Swiss eHealth strategy [36]

This national strategy aims to foster the process of digitalisation in health, thus increasing the quality of care and patient safety. The law on electronic health records (EHRs) was adapted concomitantly [37]. Although EHRs are now mandatory in hospitals and nursing homes, adhesion to EHRs in the outpatient sector is left up to patients and their healthcare professionals. Additionally, the establishment of EHRs is facing resistance to digitalisation and technical challenges linked to the variety of existing medical softwares [38], which calls for improved standardisation and interoperability [39].

### 2019: Supporting cantons’ moves towards more IC [40]

To celebrate its 100th anniversary, the Conference of Cantonal Health Directors commissioned a *Guide to Integrated Care* [40] providing cantons with information on the basic principles of IC, suggestions on implementation and a checklist for assessing the feasibility of IC at the cantonal level.

### 2020–2030: Federal Council’s health strategy [41]

Building on 2013’s National Health Strategy and lessons learned from the policies above, the 2020–2030 strategy has eight main objectives: i) exploit health data and technologies; ii) strengthen health literacy; iii) make more professionals and funding available; iv) enable healthy ageing; v) increase the quality and coordination of care; vi) control costs and their burden on deprived households; and improve health through better vii) natural and viii) work environments. This strategy promotes “coordinated care” - a synonym for IC in Switzerland - through various policy initiatives at the micro (between providers), meso (between organisations) and macro levels (between federal, cantonal and local stakeholders).

## Advances in IC in Switzerland

Whereas the previous section reflects the cautious but steady calls for IC across Switzerland’s healthcare system, this section describes the actual advances in the implementation of IC.

A national survey carried out in 2015 and 2016 aimed to produce a comprehensive overview of IC initiatives in Switzerland [18]. The survey evaluated initiatives fulfilling the following four criteria: i) a formalisation of IC principles (e.g. an agreement between several organisations, a public mandate, a research protocol, a report); ii) the integration of at least two levels of healthcare services (e.g. physician-led primary care, non-physician-led primary care, homecare services, community services, inpatient services); iii) the integration of at least two different groups of healthcare professionals (e.g. physicians, nurses, pharmacists, physiotherapists, social workers, volunteers, informal carers); and iv) the initiative was ongoing during the survey period.

The survey allocated the 155 initiatives identified to one of the six following categories: i) *Specific groups* (e.g. somatic disease-specific, 33% of the initiatives); ii) *Mental health* (26%); iii) *Coordination of care* (16%); iv) *Healthcare centres* (13%); v) *Physicians’ networks* (6%); and vi) *Drug management* (5%). Findings showed that the implementation of IC had accelerated over the past three decades. Indeed, whereas half of the 155 initiatives had started between 1990 and 2009, the other half had started between 2010 and 2016. The largest increase was found in the *Specific groups* and *Mental health* categories, two types of initiatives that require strong coordination and integration. Moreover, whereas *Specific groups* initiatives increased 3.5-fold between 1996 and 2009, but only 2-fold between 2010 and 2016, *Coordination of care* initiatives increased 4-fold and 4.2-fold, respectively. This might reflect an emerging shift towards initiatives fostering coordination of care processes instead of disease-centred initiatives.

The survey also showed that until 2012, most initiatives occurred in Switzerland’s German-speaking regions. However, by 2016, 52% were being implemented in French- and Italian-speaking regions, 45% were in German-speaking regions, and 3% were across Switzerland. Furthermore, the types of IC initiatives were different between the regions. Indeed, German-speaking regions had more *Physicians’ networks* and *Healthcare centre* initiatives, whereas in French- and Italian-speaking regions had more initiatives targeting *Specific groups, Drug management* and the *Coordination of care*. The range of professionals involved in initiatives varied too. The broadest spectrum was found in *Healthcare centre* initiatives (seven types of professionals), followed by *Specific group* initiatives (six types of professionals).

Secondary data analyses of this national IC survey explored the influences of organisation and the funding of care on the implementation of interprofessional collaboration. Findings suggested that financial barriers hindered the association of interprofessional collaboration and organisational improvements [42].

A more recent nationwide survey showed that the Swiss healthcare system’s readiness for IC could be improved [43]. Indeed, seven of the SCIROCCO tool’s [44] twelve items were rated as low (*Readiness to Change, Structure and Governance, Standardisation and Simplification, Funding, Breadth of Ambition, Innovation Management* and *Capacity Building*). Results also varied slightly, with German-speaking respondents giving lower ratings to three items (*Population Approach, Citizen Empowerment* and *Evaluation Methods*) than did French- and Italian-speaking respondents and higher ratings to *Readiness to Change* and Structure and Governance. Furthermore this survey highlighted several barriers to IC at two levels: at the professional level (e.g. threat to financial benefits) and at the system level (lack of support and training for professionals, lack of political will) [45].

## Future challenges and opportunities

Health patterns worldwide and in Switzerland have been changing [5,46]. Indeed, not only has the prevalence of chronic diseases such as cancer, diabetes, COPD, asthma and depression increased significantly, but many people have multiple coexisting (chronic) somatic and mental health conditions (multimorbidity) as well as social needs [3]. In spite of this epidemiological situation, data about the distribution of care provision in Switzerland is scarce [47], making it difficult to assess the numbers and specialities of the professionals involved in patient care or their degree of collaboration. However, in light of the increasing financial burden and number of annual medical consultations per capita, we may wonder whether Swiss patients face risks of information loss or discrepancies, potential concomitant over-investigation, over-treatment, complications, emergencies and rehospitalisation, with their concurrent negative effects on patient outcomes and costs [3,48,49,50].

These elements call out for improvements to IC. However, its potential to improve efficiency, patient safety and the quality of outcomes is only being exploited minimally in Switzerland, despite the various policies previously described showing that IC is acknowledged by growing numbers of stakeholders at the national, cantonal and local levels. This can probably be explained by Switzerland’s federal system [51]. From a change management perspective [52], this federal, decentralised system enables ad hoc innovations to be triggered by local leaders of change. However, it also leaves space for inertia and resistance to change. In order to implement IC more broadly, all across the country and in its various local contexts, the Swiss healthcare system needs robust health system building blocks [9,53]. These building blocks include initial and continuous education addressing IC both for practitioners and managers [34], adjusted financing schemes promoting coordination [54], and interoperable clinical information tools [36]. While most of these building blocks have emerged already, they will only stabilise and spread with a subtle combination of centralized and decentralized impulses. In addition to these building blocks, proactive change management strategies should make IC easy and desirable, not only to early adopters, but also to the majority of the country’s healthcare stakeholders [52,55]. Among them, healthcare system users should be more explicitly included as their perspectives would reinforce the relevance and desirability of IC [56]. While direct democracy enables lobbies such as health insurers or health professionals to have representatives at the federal level, the patients’ lobby remains poorly represented and still lacks power of action in Switzerland. However, whereas the patient-as-partners approach has been adopted at the international level (e.g. Canada [57]), care institutions in Switzerland have only recently included formal patient expertise in their governance (e.g. Geneva [58]). Finally, supporting IC in Switzerland will also contribute to its health system performance, as advocated for by the successive Triple to Quintuple Aim approaches [59,60,61,62]. However, due to the lack and/or the opacity of data (e.g. quality of services, negotiation of payment rates) [17,47], assessing this performance remains a challenge.

Against this background, the most recent developments regarding IC in Switzerland are of particular interest. Indeed, an adaptation of the MHI law is currently going through Switzerland’s democratic discussion process [63], and it would definitely support the development of IC in Switzerland:

- A mandatory first point of contact: all individuals would choose a first point of contact (e.g. physician, call centre, group practice or coordinated care network) for advice, treatment prescription or referral to another healthcare provider; the first point of contact would receive a flat-rate payment for each insured patient; exceptions to this mandatory contact would include emergency and gynaecological consultations.
- Coordinated care networks: these networks would bring together professionals from different disciplines to coordinate the entire care process; all services—including coordination activities—would be covered by a flat-rate payment.
- Patient care programmes: more evidence-based, structured processes of care—such as disease-management, prevention or rehabilitation programmes—would be developed; patients would be included in such initiatives more systematically.

These three measures would bring major changes to Switzerland’s healthcare system. Indeed, they would i) recognize the worth and importance of coordination activities, ii) partly replace fee-for-service payments by flat-rate payment systems, and iii) mitigate the central role of single physicians and facilitate care and case-management activities by a range of professionals. Because of Switzerland’s democratic processes, these measures are still far from implementation. Indeed, the dichotomy between centralised control and decentralised autonomy—representing the extremities of Switzerland’s spectrum of political combinations—has long been the subject of debate both inside and about the country’s health systems [64,65]. The dichotomy relies on two main aspects. Firstly, in the West, health is generally understood to be a public good requiring strong central leadership. Secondly, the importance of personal responsibility for one’s own health calls for reduced public involvement [66]. In Switzerland, this configuration and the various strengths and weaknesses discussed in this article offer interesting material with which to explore the diverse developments in healthcare in general and IC in particular.

## Conclusion

The number of local integrated care (IC) initiatives in Switzerland has been steadily growing over the last 20 years. With a certain lag, momentum in favour of IC policies has developed. In the future, Switzerland’s healthcare system will probably navigate between centralised support for IC and scattered local IC initiatives. This will be the reflection of a very Swiss way forward in a world without much clear evidence of whether centralised (state) incentives or decentralised (field) initiatives are more successful at developing and scaling up IC.
